# A Venue-Based Survey of Malaria, Anemia and Mobility Patterns among Migrant Farm Workers in Amhara Region, Ethiopia

**DOI:** 10.1371/journal.pone.0143829

**Published:** 2015-11-30

**Authors:** Rebekah Stewart Schicker, Neway Hiruy, Berhanu Melak, Woyneshet Gelaye, Belay Bezabih, Rob Stephenson, Amy E. Patterson, Zerihun Tadesse, Paul M. Emerson, Frank O. Richards, Gregory S. Noland

**Affiliations:** 1 Department of Global Health, Rollins School of Public Health, Emory University, Atlanta, Georgia, United States of America; 2 Nell Hodgson Woodruff School of Nursing, Emory University, Atlanta, Georgia, United States of America; 3 The Carter Center, Addis Ababa, Ethiopia; 4 Amhara Regional Health Research Laboratory Center, Bahir Dar, Ethiopia; 5 Amhara National Regional State Health Bureau, Bahir Dar, Ethiopia; 6 Department of Health Behavior and Biological Sciences, School of Nursing and The Center for Sexuality and Health Disparities, University of Michigan, Ann Arbor, Michigan, United States of America; 7 The Carter Center, Atlanta, Georgia, United States of America; 8 Agnes Scott College, Public Health Department, Decatur, Georgia, United States of America; 9 International Trachoma Initiative, Task Force for Global Health, Atlanta, Georgia, United States of America; Centro de Pesquisa Rene Rachou/Fundação Oswaldo Cruz (Fiocruz-Minas), BRAZIL

## Abstract

**Background:**

Mobile populations present unique challenges to malaria control and elimination efforts. Each year, a large number of individuals travel to northwest Amhara Region, Ethiopia to seek seasonal employment on large-scale farms. Agricultural areas typically report the heaviest malaria burden within Amhara thereby placing migrants at high risk of infection. Yet little is known about these seasonal migrants and their malaria-related risk factors.

**Methods and Findings:**

In July 2013, a venue-based survey of 605 migrant laborers 18 years or older was conducted in two districts of North Gondar zone, Amhara. The study population was predominantly male (97.7%) and young (mean age 22.8 years). *Plasmodium* prevalence by rapid diagnostic test (RDT) was 12.0%; One quarter (28.3%) of individuals were anemic (hemoglobin <13 g/dl). Nearly all participants (95.6%) originated from within Amhara Region, with half (51.6%) coming from within North Gondar zone. Around half (51.2%) slept in temporary shelters, while 20.5% regularly slept outside. Only 11.9% of participants had access to a long lasting insecticidal net (LLIN). Reported net use the previous night was 8.8% overall but 74.6% among those with LLIN access. Nearly one-third (30.1%) reported having fever within the past two weeks, of whom 31.3% sought care. Cost and distance were the main reported barriers to seeking care. LLIN access (odds ratio [OR] = 0.30, *P* = 0.04) and malaria knowledge (OR = 0.50, *P* = 0.02) were significantly associated with reduced *Plasmodium* infection among migrants, with a similar but non-significant trend observed for reported net use the previous night (OR = 0.16, *P* = 0.14).

**Conclusions:**

High prevalence of malaria and anemia were observed among a young population that originated from relatively proximate areas. Low access to care and low IRS and LLIN coverage likely place migrant workers at significant risk of malaria in this area and their return home may facilitate parasite transport to other areas. Strategies specifically tailored to migrant farm workers are needed to support malaria control and elimination activities in Ethiopia.

## Introduction

Malaria is a life-threatening parasitic disease that is transmitted to humans through the bite of an infected *Anopheles* mosquito. The disease is both preventable and treatable, yet annually there are an estimated 207 million cases and 627,000 deaths globally, with the vast majority of cases (80%) and deaths (90%) occurring in sub-Saharan Africa [1]. In Ethiopia, the second most populous country in Africa, around 68% of its 84 million people are at risk for malaria [2]. Both *Plasmodium falciparum* and *Plasmodium vivax* are prevalent and malaria is a leading cause of outpatient visits, hospitalizations and deaths across the country [2]. In 2005, Ethiopia embarked on a major scale-up of malaria prevention and control measures including: distribution of long-lasting insecticidal nets (LLINs), targeted indoor residual spraying (IRS) of insecticide, use of rapid diagnostic tests (RDTs) for confirmatory diagnosis of malaria, and introduction of artemisinin-based combination therapy (ACTs). These interventions have helped to reduce the national prevalence of *Plasmodium* infection to less than 1% in recent national surveys [3, 4]. However malaria transmission in Ethiopia occurs with significant spatial, seasonal, and inter-annual variation [5–7]. Importantly, the intervention unit for IRS and LLINs as well as indicators used to measure their coverage, are household-oriented. Thus there is substantial risk that pastoralists, refugees, migrants and others who do not physically reside within permanent households may be overlooked by conventional intervention strategies and also omitted from standard household surveys.

Recent studies have shown that history of travel is a risk factor for malaria in parts of Ethiopia [8, 9]. One of the major forces driving macro-level population movements is seasonal agricultural labor [10]. Agriculture is a key industry, accounting for around half of Ethiopia’s GDP, 80% of exports and 80% of total employment [2]. Vast portions of fertile lowland in northwest Ethiopia have been developed for large-scale agricultural farming of cash crops such as sorghum, sesame and cotton. It is estimated that annually at least 350,000 individuals travel to areas of North Gondar zone, along the border with Sudan, to seek temporary employment on such farms. As both farming and malaria transmission are inextricably linked to rainfall, the agricultural season (June to October) overlaps with the main “*kiremt*” rains (July to September) and the subsequent major malaria transmission season (September to December), placing farm workers at significant risk of malaria. This may be exacerbated by recent observations that surface irrigation schemes are associated with increased abundance of *Anopheles arabiensis*, the primary malaria vector in Ethiopia, higher sporozoite rates and greater malaria incidence in Ethiopia [11, 12]. Within Amhara Region (population 19.2 million), North Gondar zone accounted for 20.9% of all confirmed malaria cases reported in Ethiopian fiscal year 2005 (July 2012—June 2013), with the districts (*woredas*) of West Armachiho and Metema reporting two of the top five highest weekly malaria incidence rates among the 166 districts of Amhara [13]. Furthermore, it is believed that many of the migrants originate from higher elevation areas with lower or absent malaria transmission. Migrants with untreated or subclinical infections may facilitate parasite transmission to other parts of the country or even import parasites into malaria-free areas. However, current information regarding malaria prevalence and malaria associated risk factors among seasonal migrant populations in northwest Ethiopia are lacking.

While the role of mobile populations in malaria transmission has been previously recognized [14], current malaria elimination efforts have focused increasing importance on migrants and other hard-to-reach populations [15–17]. This is highlighted by the recent Victoria Falls Declaration, which articulates a specific commitment to addressing the challenges posed by mobile populations to malaria control and elimination [18]. One of the biggest challenges is use of appropriate survey methods to assess these groups. Traditional population-based surveys, such as the Malaria Indicator Survey (MIS), that utilize household sampling strategies are unlikely to account for dynamic populations or those not living in formal households. Innovative sampling approaches are thus required. Venue-based sampling, a form of time-space sampling or time-location sampling, is an alternative strategy that provides a framework to obtain a representative sample of a target population at specific times and locations [19–21]. It has most frequently been utilized in HIV research of men who have sex with men (MSM) [22–25] and illicit drug users [26, 27]. Here, for the first time in the context of malaria research, we applied a venue-based sampling strategy to investigate the demographics, prevalence of malaria and anemia, and access to, coverage and use of malaria prevention and treatment measures among migrant farm workers in northwest Ethiopia.

## Materials and Methods

### Study Area

**Fig 1:**
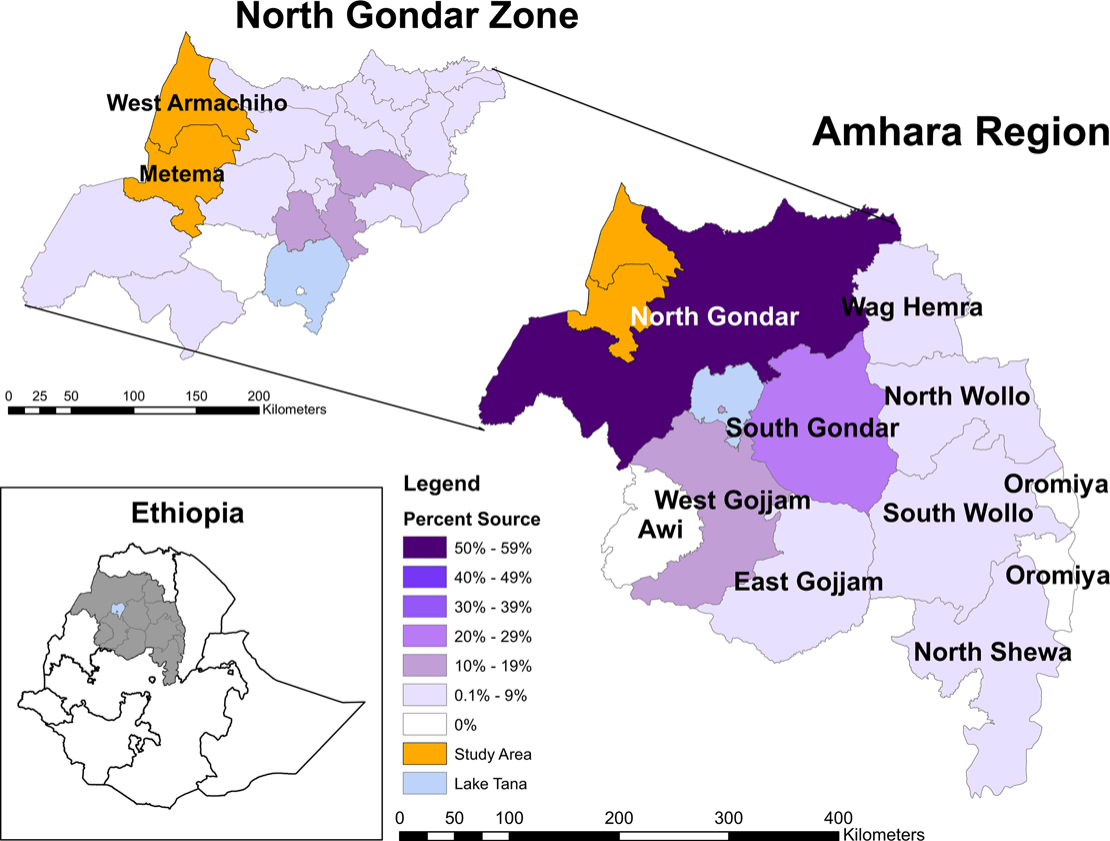
Reported Home Areas. Distribution of reported home areas within Amhara Region (lower right) and North Gondar Zone (upper left) among migrant farm workers in Metema and West Armachiho districts, Ethiopia, July 2013.

This survey was conducted in the adjoining districts of Metema (permanent resident population: 122,000; elevation: 717m) and West Armachiho (permanent resident population: 36,000; elevation: 652m) in Amhara Region, Ethiopia (Fig 1). The two districts were considered as a single survey domain. The survey took place 17–26 July, 2013 at the beginning of the farming season and immediately prior to the start of the rains that year.

### Study Design and Venue Selection

The design was a cross sectional survey employing a venue-based sampling strategy. This strategy requires initial formative research to identify locations and times in the study area that migrant farm workers could be contacted [21]. Study teams gathered information from officials at the Bureau of Environmental Protection, Land Administration and Use, Bureau of Labor and Social Affairs, health officials at regional, zonal and woreda levels, and Médecins Sans Frontières staff working in the study area. Site visits to local farms were also conducted, where discussions were held with farm managers and workers. Based on this formative research, three types of venues were identified as locations where migrant farm workers were likely to be found: farms, roads between farms, and town centers in the study area.

Venue 1: Farms. The sampling frame for farms consisted of a list of 104 “blocks” of land in the study area allocated for investor farm development obtained from the Regional Bureau of Environmental Protection, Land Administration and Use. A block encompasses approximately 1000 hectares in Metema and 1500 hectares in West Armachiho. A block could contain as many as 30 small farms or as few as one, or even a part of one, large farm. From this list, a total of 60 blocks were randomly selected. This number was predicted to yield the desired number of survey participants based on formative research results. Within selected blocks, survey teams approached the first farm that they encountered after entering the block. If individuals were present at that farm (working or otherwise), the teams would approach that group of persons and randomly choose 33% of them for eligibility screening. Selection fractions for each venue type were pre-determined based on approximate number of individuals present at each venue during formative research to achieve an approximate survey sample size of 200 persons per venue type (farms, roads, towns). Once individuals were sampled within a block, no further farms within that block were approached or sampled. If individuals were not present, the teams would move to an adjacent farm within the block until they either found individuals to sample or visited all of the farms within that selected block. If individuals were not found within the entire block at the day of sampling, the block was considered to have no migrants and was not revisited. Blocks without individuals were not replaced for sampling.

Venue 2: Roads. The same 60 blocks selected for farm sampling were also used for sampling at road venues. Within selected blocks, teams would also approach the first individuals that they encountered walking along the road in between farms and randomly select 33% of them (minimum of 3 individuals or all individuals if fewer than 3) for eligibility screening. If no migrants were found walking within an hour of arriving at the roadside within the selected block, then that venue was not revisited, not replaced, and the absence of individuals walking along the road within that block was documented.

Venue 3: Town Centers. Due to the unseasonably late arrival of rains and concern that migrants would not be found in sufficient number on farms at the time the survey would be conducted, town centers were included as a venue type. Formative research revealed that migrants openly congregate in large groups in town centers before being hired or dispersing to farms to seek employment. Ten towns were identified within the study area and all ten towns were included for sampling. At each town, study teams went to town centers and located the nearest group of openly assembled individuals appearing to be waiting for farm hire. All persons in the group were enumerated and 15% of them were approached for eligibility screening.

### Data Collection

Selected individuals were taken aside for confidential eligibility screening, data collection and blood testing. All migrants 18 years of age or older who worked, or intended to work, on a farm in the study area at the time of the survey were eligible for inclusion. A migrant was defined as a non-resident of the study area who had traveled there within the previous six months.

Survey data were collected from consenting participants via an electronic, Amharic-language questionnaire created with SwiftInsights survey software [28] and loaded onto Android-based hand-held tablet computers (Samsung GalaxyTab2 7.0). The questionnaire and sampling procedures were pre-tested prior to the start of the survey. Each interview took approximately 15 minutes and was conducted in Amharic by trained data collectors.

### Survey population

A total of 639 persons from 93 venue locations (48 farms, 35 roads, 10 towns) were selected for inclusion in the survey. An additional 37 venues (12 farms, 25 roads), were visited at which no migrant workers were found. Of selected individuals, one (0.2%) declined to participate, while 33 (5.2%) persons did not meet inclusion criteria (15 persons under the age of 18 years and 18 persons because they had been in the study area longer than 6 months). This resulted in a final sample population of 605 persons: 352 (58%) from farms, 157 (26%) from towns, and 96 (16%) from road venues.

### Blood Testing

Survey participants were also offered blood testing for malaria and anemia. Consenting individuals were tested on-site by certified laboratory technicians for malaria by RDT (SD Bio-Line Malaria Ag P.f./P.v. POCT, Standard Diagnostics, Inc.) and anemia by handheld spectrophotometer (HemoCue Hb 201+). RDT testing was conducted according to manufacturer’s instructions, and individuals with positive RDT results were offered on-site treatment according to national guidelines: artemether-lumefantrine (Coartem, Novartis AG) for non-pregnant individuals infected with *P*. *falciparum* or mixed infection, or chloroquine for *P*. *vivax* infections. Anemia was classified according to WHO guidelines [29]: normal (hemoglobin [Hb] ≥ 13 g/dL), mild (Hb 11.0–12.9 g/dL), moderate (Hb 8.0–10.9 g/dL), and severe (Hb < 8.0 g/dL). Anemic individuals were referred to the nearest health facility for further evaluation and treatment.

### Sample Size Determination

The survey was powered to detect a prevalence of malaria of 10% with absolute precision of +/-2.5% at the 95% significance level (two-sided), and 10% non-response. This yielded a target sample size of 608 individuals.

### Data Analysis

All tablet-collected survey data were downloaded to a laptop computer and exported into Excel. Descriptive univariate frequencies and proportions were calculated. Chi-squared tests were used to assess associations between categorical variables and Student’s t-tests used to compare differences in means of continuous variables. P-values <0.05 were considered statistically significant. Univariate logistic regression analysis was conducted to examine the association between RDT-diagnosed *Plasmodium* infection and various travel, work and other potential explanatory variables. Stata v13 (Stata Corp., College Station, TX) was used for all analysis.

### Ethics Statement

Participation in this survey was voluntary and anonymous; names or other personal identifiers of participants were not recorded. Only individuals 18 years of age or older were eligible to participate. Consent discussions were conducted in the local language (Amharic) and hard-copy versions of the consent script (Information Sheets) were made available to participants. Participants could refuse to answer any question or stop the interview at any time. Participants were not compensated for their participation. Individual oral consent was obtained and recorded on the tablet computers prior to the interview and blood testing. Oral consent was obtained in place of written consent due to expected high rate of illiteracy among the target population. Oral consent was also obtained from farm managers and workers for preliminary site visits, but was not recorded due to the informal nature of the discussions. The entire survey, including protocol, data collection instrument and consent procedures, was approved by the Ethical Review Committee of Amhara National Regional State and Emory University’s Institutional Review Board (protocol IRB00066825).

## Results

### Sample Demographics

Of 605 surveyed individuals, nearly all (97.7%) were male (Table 1). Mean age was 22.8 years (standard deviation [SD] 6.5 years; range 18–65 years), with the majority (74.1%) less than 25 years of age. The most common ethnicity was Amhara (96.0%). Around half (48.4%) of respondents were able to read a full sentence in Amharic. Parts of an Amharic sentence could be read by 12.9% of respondents, while 38.4% could not read at all. More than one-third (36.0%) of participants reported that they traveled to the study area with a family member. Of these, niece/nephew was the most common (51.8%) response followed by siblings (30.7%). The majority (72.9%) of participants were currently employed in paid farm work at the time of survey. Not surprisingly, a greater proportion of those surveyed on farms (81.7%) were already working compared to those on roads (53.1%) and in towns (66.2%).

**Table 1:** Characteristics of surveyed migrant farm workers in Metema and West Armachiho districts, Amhara Region, Ethiopia, July 2013 (N = 605).

		N (%)
**Sex**	Male	591 (97.7)
	Female	6 (1.0)
	Missing	8 (1.3)
**Age (years)**	18–19	201 (33.2)
	20–24	247 (41.0)
	25–29	97 (16.0)
	30–34	21 (3.5)
	35–65	34 (5.6)
	Missing	5 (0.8)
**Ethnicity**	Amhara	581 (96.0)
	Tigre	17 (2.8)
	Oromo	3 (0.5)
	Sudanese	1 (0.2)
	Other	2 (0.3)
	Missing	1 (0.2)
**Ability to read Amharic**	Can read whole sentence	293 (48.4)
	Can read only parts of a sentence	78 (12.9)
	Cannot read sentence at all	232 (38.4)
	Blind/visually impaired	0 (0.0)
	Missing	2 (0.3)
**Accompanied family member(s)**	Yes	218 (36.0)
	No	384 (63.5)
	Missing	3 (0.5)
**Relationship of traveling family member(s)**	Niece or Nephew	113 (51.8)
(multiple responses possible)	Siblings	67 (30.7)
	Aunt or Uncle	20 (9.2)
	Children	5 (2.3)
	Parents	4 (1.8)
	Spouse	2 (0.9)
	Other	23 (10.6)
**Started paid farm work**	Yes	441 (72.9)
	No	162 (26.8)
	Missing	2 (0.3)

### *Plasmodium* Prevalence

Results for malaria testing by RDT were available from 592 (98%) of survey participants. Prevalence of *Plasmodium* infection was 12.0% overall: 9.6% *P*. *falciparum*, 1.7% *P*. *vivax*, and 0.7% mixed infections (Table 2). The relative species proportions were 80% *P*. *falciparum*, 14% *P*. *vivax*, and and 6% mixed. There was not a significant difference in RDT positivity between age groups (*P* = 0.49), the three different venue types: farms (11.6%), roads (11.1%) and towns (12.4%; *P* = 0.95), the two survey districts: Metema (10.9%) and West Armachiho (12.2%; *P* = 0.65), or arrival time: July (10.2%) versus earlier (12.7%; *P* = 0.38). Nearly one-third (30.1%) of all survey participants reported having a fever within the preceding two weeks and there was a significant association between RDT positivity and reported fever (*P* = 0.01), as 17.2% of those reporting fever were RDT-positive versus 9.4% of those not reporting fever in the past two weeks.

**Table 2:** Species-specific *Plasmodium* infection, diagnosed by rapid diagnostic test (RDT), among migrant farm workers, Metema and West Armachiho districts, Amhara Region, Ethiopia, July 2013 (N = 592).

	N (%)
*Plasmodium falciparum (Pf)*	57 (9.6)
*Plasmodium vivax (Pv)*	10 (1.7)
Mixed *Pf*—*Pv*	4 (0.7)
RDT Negative	521 (88.0)

### Anemia Prevalence

Results from anemia testing were available from 569 (94%) of survey participants. Mean hemoglobin (Hb) of the tested individuals was 13.8 g/dl (SD = 1.8; range 7.2–18.7). Overall prevalence of anemia (Hb < 13 g/dl) was 28.3% (Table 3), with 22.9% of individuals diagnosed as mildly anemic (Hb 11.0–12.9 g/dl), 5.3% moderately anemic (Hb 8.0–10.9 g/dl), and 0.2% severely anemic (Hb <8 mg/dl) using WHO thresholds [29]. Anemia was significantly associated with malaria infection (*P* < 0.001), as more than half (52.9%) of RDT-positive individuals were diagnosed with anemia, compared to 25.2% of those with negative RDT result. Mean hemoglobin values were significantly different between RDT-positive (12.7 g/dL) and RDT-negative (14.0 g/dL) individuals (*P* < 0.001). Prevalence of anemia was not significantly associated with reported fever in the past two weeks (*P* = 0.90).

**Table 3:** Anemia prevalence, by severity, among migrant farm workers, Metema and West Armachiho, Amhara Region, Ethiopia, July 2013 (N = 569).

	N (%)
Normal (hemoglobin [Hb] ≥13.0 g/dl)	408 (71.7)
Mild anemia (Hb 11.0–12.9 g/dl)	130 (22.9)
Moderate anemia (Hb 8.0–10.9 g/dl)	30 (5.3)
Severe anemia (Hb < 8.0 g/dl)	1 (0.2)

### Travel History

The majority (79.3%) of survey participants reported that they have a “home area” (defined as an area to which they return to at least once a year when not engaged in paid farm work elsewhere). Nearly all (95.6%) of those who identified a home area came from within the Amhara Region (Fig 1). Of those from Amhara Region, more than half (51.6%) identified North Gondar as their home zone, followed by South Gondar (27.9%) and West Gojam (12.9%, including 3.7% from Bahir Dar Town). Of those from North Gondar Zone, nearly half (45.6%) of migrants came from three woredas that surround the town of Gondar approximately 100–150 km away from the study area: Dembia (18.1%), Gondar Zuria (14.4%) and Wogera (13.1%).

As shown in Table 4, the majority (62.7%) of survey participants who designated a home area stated that they usually spend more than six months per year there. Most (86.1%) said they came to the study area directly from their home area. The majority of respondents reported arriving into the study area in either June (39.2%) or July (37.7%), though some arrived as early as January. Public transportation (bus, truck, *bajaj* [three-wheel motorized taxi], hired car or hired motorcycle) was the most commonly reported method of transportation to the study area (98.6%), followed by walking (66.2%). Other less common methods are reported in Table 4. A majority of respondents (78.8%) reportedly worked in the study area at least one previous year. Only 22.8% of migrants reported working for pay in other farm locations outside the study area in the past twelve months. When asked what month they intended to leave the study area, almost half planned to leave in September (45.6%), with the majority (84.8%) indicating they would return to their home area.

**Table 4:** Migration patterns of migrant farm workers in Metema and West Armachiho districts, Amhara Region, Ethiopia, July 2013 (N = 605).

		N (%)
**Month of arrival in study area**	January	3 (0.5)
	February	6 (1.0)
	March	13 (2.2)
	April	24 (4.0)
	May	92 (15.2)
	June	237 (39.2)
	July	228 (37.7)
	Missing	2 (0.3)
**Transportation method to study area**	Public transportation	597 (98.6)
(multiple responses possible)	Walking	401 (66.2)
	Tractor	69 (11.4)
	Owned car/motorcycle	2 (0.3)
	Animal/animal cart	2 (0.3)
	Missing	3 (0.5)
**Most recent location prior to arrival in study area**	Home area	520 (86.1)
	Not home area	68 (11.2)
	No home area identified	11 (1.8)
	Missing	6 (1.0)
**Month of planned departure from study area**	August	33 (5.5)
	September	276 (45.6)
	October	60 (9.9)
	November	56 (9.3)
	December	54 (8.9)
	January	43 (7.1)
	February—April	5 (0.8)
	More than one year	8 (1.3)
	No intention to leave	9 (1.5)
	Don’t know	52 (8.6)
	Missing	9 (1.5)
**Departure destination**	Home area	513 (84.8)
	Not home area	60 (9.9)
	No home area identified	10 (1.7)
	Don’t know	14 (2.3)
	Missing	8 (1.3)
**Number of times to study area for paid farm labor**	Never	125 (20.7)
	Once	115 (19.0)
	Twice	101 (16.7)
	Three times	91 (15.0)
	More than three times	170 (28.1)
	Missing	3 (0.5)
**Performed paid farm work outside of study area in previous 12 months**	Yes	138 (22.8)
	No	459 (75.9)
	Don’t know	2 (0.3)
	Missing	6 (1.0)
**Usual time spent in home area** †	< 6 months	176 (36.7)
(If a home area identified, N = 480)	> 6 months	301 (62.7)
	Missing	3 (0.6)

†‘Home area’ defined as an area to which participants return to at least once a year when not engaged in paid farm work elsewhere.

### Malaria Exposure Factors and Prevention Measures

**Fig 2:**
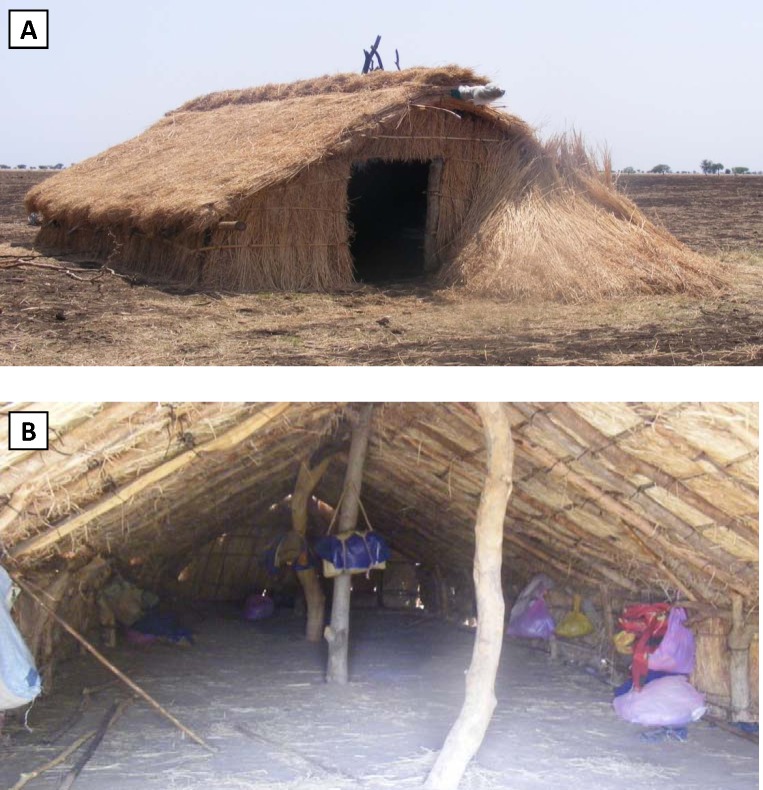
Typical Farmer’s Shelter. Exterior (A) and interior (B) of typical temporary shelters used by seasonal farm workers in northwest Ethiopia.

The majority of respondents (51.2%) reported a temporary shelter as their usual sleeping accommodation (Table 5). On farms, these are typically thatch structures constructed by the migrants during the farming season that can house up to 100 persons in very close quarters (Fig 2). However, ‘temporary shelter’ also includes any type of non-permanent structure used in towns or on farms. Approximately one-fifth of respondents indicated sleeping outside (20.5%) or in a house (18.2%). Within the various venue-types, temporary shelter was the most commonly reported accommodation for those on farms (64.2%) and on roads between farms (51.1%), while town respondents were more evenly distributed between temporary shelters (25.0%), houses (25.0%) and hotels (17.8%). Sleeping outside was more common among those on roads (25.5%) and in towns (26.3%) versus those on farms (17.2%), likely reflecting the established nature of existence for those who had secured farm employment.

**Table 5:** Malaria exposure and prevention measures among migrant farm workers, Metema and West Armachiho districts, Amhara Region, Ethiopia, July 2013 (N = 605).

		N (%)
**Usual sleeping accommodation**	Temporary shelter	310 (51.2)
	Outside	124 (20.5)
	House	110 (18.2)
	Hotel/Dormitory	33 (5.5)
	Tent	2 (0.3)
	Other	16 (2.7)
	Missing	10 (1.7)
**Accommodation sprayed with IRS in the past 12 months**	Yes	5 (3.5)
(if accommodations are a house or hotel/dormitory, N = 145)	No	58 (40.0)
	Don’t know	82 (56.6)
**LLIN available**	Yes	72 (11.9)
	No	528 (87.3)
	Missing	5 (0.8)
**Own the LLIN**	Yes	44 (61.1)
(If LLIN available, N = 72)	No	28 (38.9)
**Where LLIN obtained**	In study area	52 (72.2)
(If LLIN available, N = 72)	Outside the study area	18 (25.0)
	Missing	2 (2.8)
**Source of LLIN**	Farm employer	30 (42.0)
(If LLIN available, N = 72)	Private shop/market	15 (21.0)
	Health post/center	14 (19.4)
	Family/friend	5 (7.0)
	Mass distribution	3 (4.2)
	Private clinic	1 (1.4)
	NGO	1 (1.4)
	Other	2 (2.8)
	Missing	1 (1.4)
**Used LLIN last night**	Yes	53 (73.6)
(If LLIN available, N = 72)	No	18 (25.0)
	Missing	1 (1.4)
**Work outside after dark at least sometimes**	Yes	64 (10.6)
	No	394 (65.1)
	Not yet working	135 (22.3)
	Missing	12 (2.0)

Of those living in a permanent housing structure (house or hotel/dormitory) the majority (56.6%) did not know if that structure had been sprayed with IRS in the past 12 months, while 40.0% reported that the structure had not been sprayed. Only 3.5% of respondents reported that the structure had been sprayed with IRS.

Only 12% of migrants reported having access to a LLIN while working here, of whom 61.1% reportedly owned the net (Table 5). Of those who had access to a net, the majority (72.2%) obtained the net in the study area, with 42.0% reporting they obtained the net from their farm employer, while around one-fifth obtained their LLIN from either a private shop or market (21.0%) or a health post or health center (19.4%). Net use was significantly higher among those who had started working (14.2%) versus those not working (6.2%; P = 0.007). Reported net use among those who had access to an LLIN was 73.6%, or 8.8% of the total survey population. Of workers who had started farm work already, 13.6% reported that they work after dark sometimes.

### Malaria Knowledge

Nearly all (94.7%) survey participants reported having heard of malaria. Of those who had heard of malaria, almost half (48.5%) correctly identified a mosquito bite as the primary cause of malaria. Of those who had heard of malaria, more than half of respondents identified fever (65.5%), chills (55.3%), shivering (50.6%), and headache (50.1%) as the signs or symptoms of malaria, and sleeping under an LLIN was the most commonly reported (75.9%) method of protection.

### Health Care Concerns, Access and Care-Seeking Behavior

This survey also inquired about respondents’ general health care concerns, access to care, and treatment seeking behavior. Malaria was the single greatest (78.0%) health concern reported by migrant workers. Nearly one-third (30.1%) of survey participants reported having a fever within the preceding two weeks (Table 6). Of those, 31.3% reported seeking some sort of treatment for their fever, with a non-significant trend for higher treatment seeking among those sampled in towns (39.3%) versus those on farms or roads (27.3%) (*P* = 0.10). Among those who sought treatment for a fever, nearly half (45.6%) went to a government health center, while 31.6% went to a private hospital/clinic. The main reasons stated for not seeking treatment among those who had a fever included lack of financial resources/treatment too expensive (43.2%), distance to facility/no transportation available (24.8%) and illness not serious (20.8%).

**Table 6:** Fever prevalence and health care seeking behavior among migrant farm workers, Metema and West Armachiho districts, Amhara Region, Ethiopia, July 2013 (N = 605).

		N (%)
**Fever within the past two weeks**	Yes	182 (30.1)
	No	411 (67.9)
	Don’t know	8 (1.3)
	Missing	4 (0.7)
**Sought advice or treatment**	Yes	57 (31.3)
(if had fever in past two week N = 182)	No	125 (68.7)
**Source of treatment**	Government health center	26 (45.6)
(if sought treatment, N = 57)	Private hospital/clinic	18 (31.6)
	Pharmacy	5 (8.8)
	Farm Management	2 (3.5)
	Government hospital	2 (3.5)
	Traditional practitioner	1 (1.8)
	Government health post	1 (1.8)
	NGO	1 (1.8)
	Missing	1 (1.8)
**Was a blood test for malaria performed?**	Yes	30 (52.6)
(if sought treatment, N = 57)	No	26 (45.6)
	Missing	1 (1.8)
**Was a medication given to you for the fever?**	Yes	54 (94.7)
(if sought treatment, N = 57)	No	3 (5.3)
**What medications were you given for the fever?**	Paracetamol	35 (64.8)
(if given medication, N = 54, multiple responses possible)	Coartem	30 (55.6)
	Chloroquine	3 (5.6)
	Quinine	1 (1.9)
	Other non-antimalarial	17 (31.5)
	Don’t know	1 (1.9)
**Reasons for not seeking treatment**	Lack of money/Too expensive	54 (43.2)
(if had fever, but did not seek treatment, N = 125)	Distance/No transportation	31 (24.8)
	Illness not serious	26 (20.8)
	Cannot leave work	5 (4.0)
	Unsure where to go	4 (3.2)
	Other	5 (4.0)

Of those who had a fever in the past two weeks and who sought treatment, 52.6% reported that a blood test for malaria was performed, with test results communicated to 70.0% of those who were tested. The vast majority (94.7%) of survey participants who sought treatment for a fever received medication, with paracetamol (64.8%) and Coartem (55.6%) the most commonly received medications for fever.

### Malaria Risk Factor Analysis

Univariate analysis was conducted to evaluate associations between RDT-diagnosed *Plasmodium* infection and various travel, work condition and other key potential risk factors for malaria. Among examined variables (Table 7), only two were significantly associated with reduced *Plasmodium* prevalence: having access to an LLIN in the study area (odds ratio [OR] = 0.30, 95% confidence interval [CI] 0.09–0.97, *P* = 0.04) and correct identification of mosquito bite as cause of malaria (OR = 0.50, 95% CI 0.29–0.87, *P* = 0.02). Reported net use the previous night was also associated with reduced prevalence (OR = 0.16, 95% CI 0.01–1.85), though the result was not statistically significant (*P* = 0.14).

**Table 7:** Univariate logistic regression analysis of travel, work related, and other risk factors for RDT-diagnosed *Plasmodium* infection among migrant farm workers in Metema and West Armachiho, Amhara Region, Ethiopia, July 2013.

Variable	N	Odds Ratio	95% CI	P-Value
**Most recent location**				
Not home area	74			
Home area	484	1.12	0.51–2.46	0.77
**Usual time spent in home area (if a home area identified)**				
<6 months	165			
>6 months	284	0.81	0.45–1.47	0.49
**Previous travel to study area for paid farm labor**				
No	119			
Yes	441	1.00	0.53–1.88	0.99
**Performed paid farm work outside of study area in previous 12 months**				
No	432			
Yes	124	1.16	0.64–2.12	0.63
**Usual sleeping accommodation**				
Permanent	139			
Outside or temporary shelter	398	1.03	0.56–1.88	0.93
**LLIN available**				
No	487			
Yes	71	0.30	0.09–0.97	0.04*
**Used LLIN last night**				
No	18			
Yes	52	0.16	0.01–1.85	0.14
**Work outside after dark at least sometimes**				
No	364			
Yes	62	1.56	0.73–3.31	0.25
**Knows mosquito bite as cause of malaria**				
No	273			
Yes	258	0.50	0.29–0.87	0.02*

* *P* <0.05

## Discussion

Results from this venue-based survey of seasonal migrant farm workers in northwest Ethiopia revealed a young population of repeat workers that originate from nearby highland and highland-fringe areas of northwestern Amhara Region and who return to their home areas at the end of farming season; a high prevalence of malaria compared to other estimates for Amhara and Ethiopia; significant prevalence of anemia; low access to health care; and low coverage of standard malaria prevention methods (LLINs and IRS). This indicates that seasonal workers face a direct threat of malaria while in this area and that their return home may facilitate transport of parasites to areas of Amhara that otherwise may have limited malaria transmission. Development of strategies specifically tailored to address the vulnerabilities of migrant farm workers are needed in order to support malaria elimination activities in Ethiopia.

To our knowledge, this is the first time that venue-based sampling has been utilized for malaria research. Recent work from the Thailand-Cambodia border used respondent-driven sampling to study malaria among migrants [30, 31]. While respondent-driven sampling and venue-based sampling each have strengths and limitations [21], venue-based sampling is more appropriate when the target population congregates in accessible locations, as was the case with our study population. Venue-based sampling was originally developed to investigate HIV and associated risk factors among young MSM [19]—a population that was particularly underrepresented in traditional survey methods. For that reason, it has been widely used in the HIV [22–24] and illicit drug use [26, 27] research fields. Venue-based sampling provides a framework to obtain a representative sample of hard-to-reach populations for which sampling frames may not exist. The application of the venue-based method, with a clear list of venues and rules pertaining to the selection of respondents within venues, prevents the sample from becoming a simple convenience sample. However, the lack of population estimates for the target population also prevents the calculation of sample weights or clustering effects; hence the sample is presented as a non-probabilistic sample. To ensure external validity with respect to the target population, venue-based sampling requires formative research to identify all, or nearly all, the venues frequented by the target population [21]. Formative research for this study concluded that the farms, roads between farms, and towns likely captured the majority of possible venue locations. Despite this, we cannot exclude the possibility that members of the target population may not attend or have been contacted at these venues. For example, prior anecdotal reports suggested that women accompany migrant workers to serve as cooks or in other supportive domestic functions. It was surprising therefore that we only identified six females for inclusion. This suggests a need to further investigate the presence of female migrants and other potentially underrepresented or missing migrants, and to consider whether alternative approaches are needed to reach these individuals. While methods have been proposed to derive weighted estimates for venue-based and time-location sampling schemes needed to make statistically valid inferences to the target population [19, 32, 33], these methods have not been widely adapted—particularly in instances like this survey, where the primary aim is an initial description of a hard-to-reach population. Nonetheless, we believe the generalizability of the sample population to the target population is high given that nearly half (46%) of the area’s 104 farmland blocks and all ten of the area’s towns were sampled. Furthermore, inclusion bias seems minimal as only one (0.2%) of 639 selected individuals declined to participate.

Mobility and agriculture-related activities have long been implicated in transmission of malaria in Ethiopia [10, 34]. This is supported by recent case-control studies documenting that history of travel is a significant risk factor for infection [8, 9]. The annual migration of an estimated 350,000 individuals to northwest Ethiopia for seasonal agricultural employment is one of the largest regular population movements in Ethiopia. This survey was conducted in two *woredas* of northwest Amhara (West Armachiho and Metema) that together receive approximately 75–80% of all seasonal migrant workers in the region. We found that nearly all survey participants originated from within Amhara Region, with half coming from within North Gondar zone. This proximate origin is an important finding of this study in light of prior anecdotal reports that seasonal migrant workers travel from a wide range of areas across Ethiopia and from neighboring Sudan. Most of the identified home areas are located in the highlands (>2000m) or highland fringe (1750—2000m) that experience limited or low-level epidemic-prone malaria transmission [2]. It is often stated that individuals from highland areas of Ethiopia have little or no immunity to malaria [8, 10]. Studies in other parts of East Africa document reduced anti-malaria immunity in individuals from highland areas of low or unstable transmission compared to individuals from higher transmission areas [35, 36], and recent work from Ethiopia found reduced prevalence and levels of total IgG antibodies to *P*. *falciparum* blood-stage antigens among residents of a highland area (2,059 m) in Dabat *woreda*, North Gondar compared to residents in a nearby mid-elevation farming area (1,380 m) [37]. However, apart from a study by Collins and colleagues from the late 1960’s [34], comparison of anti-malaria immune responses across Ethiopian transmission gradients are lacking, and further work is needed to define susceptibility differences and immunologic correlates. As 85% of participants indicated that they would return directly to their home area, there is a potential that untreated or asymptomatic infections in migrants may enable transport of parasites to locations throughout northwest Amhara—including to migrants’ destination home areas as well as to intermediate locations where they spend the night. Genetic analysis, similar to that recently reported from Senegal [38], comparing parasites strains collected in lowland farming areas and in home areas common to migrants would provide valuable insight into the epidemiology of malaria in the region and aid geographic targeting of interventions. In addition, detailed analysis of surveillance data from identified home areas would inform development of “source-sink” maps of predicted parasite and population movements [17, 39].

This survey provides further evidence that the lowland areas of northwest Amhara experience a higher burden of malaria compared to Amhara region as a whole. The 2011 Ethiopia Malaria Indicator Survey (MIS) found 2.0% *Plasmodium* prevalence by microscopy across malarious areas (areas <2000 m) of Amhara [4], while the 2007 MIS microscopy estimate was 0.6% for areas <2500 m [3]. In contrast, we observed 12.0% *Plasmodium* infection by RDT among migrants in a lowland area of North Gondar. The higher prevalence observed here could be attributed to antigen persistence or functional sensitivity differences between RDT and microscopy, since surveys [40], including ones from Ethiopia [4, 41], frequently report higher RDT-diagnosed prevalence compared to microscopy. However, malaria is known to exhibit significant spatial, seasonal, and inter-annual variability across Ethiopia [5, 7]. Metema and West Armachiho, which comprise only 4.7% of North Gondar’s permanent population, accounted for a 26.9% of zone’s confirmed malaria cases reported in July 2012—Jun 2013 (Ethiopian fiscal year 2005) [13], and the highest burden clusters in Amhara from the 2011 MIS were located in or very close to our study area [4]. Unlike the MIS surveys of 2007 and 2011 [3, 4], which were conducted in October—December to coincide with the major malaria transmission period, this survey was conducted in July prior to the main rains. Therefore, we predict that malaria prevalence in lowland areas during peak transmission—a time when harvest and farm worker activity are also at a maximum—is higher than observed in this survey. This is supported by analysis of inpatient data from Metema hospital over a six year period documenting that total number of suspected malaria cases and the slide positivity rate peaks in the months September through November [42].

This survey also observed a lower proportion of *P*. *vivax* infections (14%) than typically reported in Ethiopia (25%-35%) [2]. This is likely explained by the greater geographic range of *P*. *vivax* in parts of the country that are inhospitable for *P*. *falciparum* sporogonic development, leading to a higher proportion of *P*. *vivax* nationwide compared to our study area. Chloroquine monotherapy is the current recommended first-line drug for *P*. *vivax* in Ethiopia [2]. Primaquine, which in combination with chloroquine was previously used to treat all *Plasmodium* infections in Ethiopia until 1990 [43], is presently recommended for radical cure of *P*. *vivax* only in non-endemic areas and available only by prescription from hospitals and health centers [2]. However, the potential for parasite dispersal with migrants and the ability of *P*. *vivax* hypnozoites to induce relapsing episodes months or years after primary infection, combined with recent evidence of minimal G6PD deficiency in Ethiopia [44], lends further weight to calls for the re-addition of primaquine for radical cure of *P*. *vivax* in all areas of Ethiopia [45] in line with WHO recommendations [46].

Prevalence of *Plasmodium* infection among participants was not significantly different between age groups, venue types, study districts or time of arrival to the study area, indicating that risk of malaria infection was fairly uniform across the sample population and study area. This also suggests that infections were acquired both outside and within the study area, though this study was not designed to determine the exact sources of *Plasmodium* infection. Information pertaining to the duration and sleeping habits of participants’ transit to the study area could help to identify additional risk factors and estimate exposure in space and time, but prospective studies are needed to determine the specific malaria risk while working on farms. Univariate analysis indicated that access to an LLIN and knowing the cause of malaria were protective against malaria, whereas RDT-positivity was not significantly associated with other examined travel, work and net-related factors. Of particular interest, reported net use the previous night was not significantly associated with reduced *Plasmodium* infection—though this finding is consistent with previous Ethiopian studies [5, 9]. This may be due to low frequency of those reporting net use the previous night. However, sporadic net use could also account for the apparent discrepancy with net availability, which highlights the need for surveys to include questions about net use patterns over a longer period of time in addition to use the previous night.

Overall mean hemoglobin in our survey was 13.8 g/dL, with significantly lower levels (12.7 g/dL) among RDT-positive individuals. This is consistent with findings from a recent clinic-based study in a rural highland area that observed differences in mean hemoglobin between malaria-uninfected (13.5 g/dL) and malaria-infected men (10.4 g/dL) [47]. More than one quarter (28.3%) of survey participants were anemic. Since the target population of migrant workers is likely subject to self-selection of a potentially younger, healthier group of individuals able to travel long distances to engage in strenuous farm labor, this estimate may not represent a population-wide estimate of male anemia in permanent residents in the study area. Such population-wide estimates of anemia in Ethiopian adult males are rare. One study determined that 44.5% of healthy adult males across a range of elevations in Gondar zone were anemic [48], while other studies of healthy adult males observed mean hemoglobin values of 15.7 g/dL [49] and 16.1 g/dL [50]—well above the WHO threshold of 13.0 g/dL for adult men [29]. Taken together, this suggests that malaria is a risk factor for anemia among adult male migrant farm workers, similar to the strong association of malaria-related anemia observed in pediatric populations in Ethiopia [51] and in hyperendemic areas elsewhere in Africa [52, 53]. However, additional studies are needed to investigate other possible etiologies, including nutritional deficiencies or other infectious diseases such as soil-transmitted helminths and schistosomiasis, in order to address the causes of anemia in adult populations, especially those in farming and other physically demanding professions.

A major objective of this survey was to assess malaria-related knowledge and utilization of current malaria prevention measures among migrant farm workers. Nearly all participants reported having heard of malaria, and knowledge of specific transmission and prevention methods was similar to that found among women in the 2011 MIS [4]. IRS was extremely uncommon, owing to the fact that only around one-quarter of respondents were living in a house or hotel/dormitory eligible for spraying, and that the majority of those housed in these structures did not have access to information as to whether the structure had been sprayed. Reported net use was high (73.6%) among those that had access to an LLIN. However, only 11.9% of survey participants had such access, meaning that overall net use in our sample population (8.8%) was significantly lower than household LLIN ownership in Amhara (73.7%) recorded in 2011 MIS [4]. We did not ask whether migrants had access to a net in their home area, or if so, why they elected to leave nets there. Since most respondents originated from highland or highland-fringe areas, and areas >2000m are not targeted for LLIN distribution in Ethiopia [2], we suspect that net availability may be equally low in home areas. Of nets available to survey participants, a majority were acquired in the study area. One-fifth of nets were purchased from a private shop or market, indicating that nets are available to migrants at a cost through the private sector. Importantly, farm employers provided nearly half (42.0%) of the available nets, with most (73%) considered the property of the employer to be returned at the end of employment. As nets are made available to employers through the Ministry of Health, greater coordination is needed between agricultural employers and the ministries of health, agriculture, and labor to ensure that more nets are provided to farm employees. However, both LLINs and IRS are ill-suited for migrant farm workers while traveling to farm areas and when employed on farms; in particular, individual LLINs are impractical to hang in the crowded multi-person layout of typical thatch shelters. Insecticide-treated plastic sheeting and durable wall lining have been successfully used in similar settings in India [54] and Papua New Guinea [55], but these products may have limited efficacy against exophilic vectors such as *An*. *arabiensis*. This, combined with the outdoor sleeping and reported night-time working patterns, suggests that vector control strategies alone will be insufficient to prevent and control malaria among migrants in western Amhara.

Few migrants have LLINs and less than one-sixth of all those with fever in the past two weeks reported being tested for malaria. Additional interventions are clearly needed that are tailored to the unique needs of migrant workers. Three such potential interventions include road-side health stations, establishment of seasonal health posts within farming areas, and mobile health teams. 1) Road-side health stations: The majority of migrants arrived to the study area on foot via a limited number of roads leading into the study areas. Road-side stations could be established during primary arrival and departure months to provide on-site testing and treatment combined with malaria and general health education. In addition to the immediate health benefits, this would also provide better data regarding malaria infection status at entry into the study area and reduce the risk of exportation from it. 2) Seasonal health posts in farming areas could provide increased access to care and fit within the existing Ethiopian health systems framework. Despite a large influx of workers during farming season, health posts are generally established based on official census figures of the permanent population. Because migrants are not considered permanent residents, health posts have not been established in farming areas. However, stakeholders should consider the benefits that seasonal health posts would offer to the large population of migrant farm workers in Amhara. 3) Mobile health teams deployed to farms during farming season could conduct mass test and treat (MTAT) activities. These teams could provide not only diagnostic and treatment services for malaria, but also help improve mass drug administration (MDA) coverage for neglected tropical diseases (NTDs) such as trachoma, onchocerciasis and lymphatic filariasis that also affect the study area. Additional strategies to the three mentioned above may be needed and interventions should be developed in consultation with stakeholders in the health, agriculture and labor sectors. Above all, it is important that interventions not lead to fear of, social stigmatization, or isolation of migrants, as this population likely already experiences a sense of vulnerability and marginalization [56].

There are important limitations to this survey, in addition to those already mentioned. This survey was conducted at the beginning of the farming season in order to collect information and assess malaria infection in a diversity of migrants, including recent arrivals and those present for longer periods of time. Nonetheless, the inclusion of a significant proportion of non-working migrants (26.8%) influenced our results in terms of the relative distribution of sleeping accommodations, net availability, time of day spent working, resource availability of survey participants, and healthcare seeking behavior. Secondly, formative research conducted during the survey indicated that there may be two waves of seasonal migrant workers to northwest Amhara: an initial younger wave that works at the beginning of the farming season during breaks in the school calendar; and a second wave of migrant laborers, thought to be older and more experienced than the first, that reportedly arrives in August to oversee the late-season harvest activities. Our results seem consistent with this, as the sample population was young, mainly arrived in June or July and planned to leave in September—when the academic year commences. There are likely important differences between these two waves of laborers not captured in our findings. Finally, the demographics, travel patterns and malaria risks of our sample population may not reflect those of seasonal migrant farmers in other areas of Ethiopia.

## Conclusion

This is the first time a formalized survey has been conducted among the migrant farm-worker population of northwestern Amhara. We observed primarily a local migrant population traveling back and forth within the same region at a time that corresponds to periods of active malaria transmission. Their prevalence of malaria is high compared with the country as a whole and the Amhara region, and their access to malaria prevention measures is low. Migrants face many barriers to accessing preventative and curative services, and may facilitate transport of malaria parasites to other areas of Amhara. We propose targeted malaria strategies for this unique population that may alleviate the burden of malaria among migrant farm workers, and aid control and elimination efforts in Ethiopia.
